# Association of socio-economic status with diabetes prevalence and utilization of diabetes care services

**DOI:** 10.1186/1472-6963-6-124

**Published:** 2006-10-03

**Authors:** Doreen M Rabi, Alun L Edwards, Danielle A Southern, Lawrence W Svenson, Peter M Sargious, Peter Norton, Eric T Larsen, William A Ghali

**Affiliations:** 1 Department of Medicine, University of Calgary, Calgary, Canada; 2 Department of Community Health Sciences, University of Calgary, Calgary, Canada; 3 Centre for Health and Policy Studies, University of Calgary, Calgary, Canada; 4 Department of Family Medicine, University of Calgary, Calgary, Canada; 5 Alberta Health and Wellness, Edmonton, Canada; 6 Calgary Laboratory Services, Calgary, Canada

## Abstract

**Background:**

Low income appears to be associated with a higher prevalence of diabetes and diabetes related complications, however, little is known about how income influences access to diabetes care. The objective of the present study was to determine whether income is associated with referral to a diabetes centre within a universal health care system.

**Methods:**

Data on referral for diabetes care, diabetes prevalence and median household income were obtained from a regional Diabetes Education Centre (DEC) database, the Canadian National Diabetes Surveillance System (NDSS) and the 2001 Canadian Census respectively. Diabetes rate per capita, referral rate per capita and proportion with diabetes referred was determined for census dissemination areas. We used Chi square analyses to determine if diabetes prevalence or population rates of referral differed across income quintiles, and Poisson regression to model diabetes rate and referral rate in relation to income while controlling for education and age.

**Results:**

There was a significant gradient in both diabetes prevalence (χ^2 ^= 743.72, p < 0.0005) and population rates of referral (χ^2 ^= 168.435, p < 0.0005) across income quintiles, with the lowest income quintiles having the highest rates of diabetes and referral to the DEC. Referral rate among those with diabetes, however, was uniform across income quintiles. Controlling for age and education, Poisson regression models confirmed a significant socio-economic gradient in diabetes prevalence and population rates of referral.

**Conclusion:**

Low income is associated with a higher prevalence of diabetes and a higher population rate of referral to this regional DEC. After accounting for diabetes prevalence, however, the equal proportions referred to the DEC across income groups suggest that there is no access bias based on income.

## Background

Socio-economic status (SES) and its constituent elements are accepted as being determinants of health. There is considerable evidence to show that poverty is associated with shorter life expectancies and increased mortality, particularly cardiovascular mortality [1-3]. Significant socio-economic gradients have been shown in the prevalence of several cardiovascular disease risk factors, including diabetes. Diabetes may be up to two times more prevalent in low income populations compared to wealthy populations [4-6]. In patients with diabetes, low income is associated with an increased rate of hospitalization for acute diabetes related complications. Booth and Hux [7] demonstrated that, even within a universal health care system, the least affluent patients were admitted to hospital 43% more often than the wealthiest patients. They also identified lack of physician directed ambulatory care as a major determinant of hospitalization in patients with diabetes.

Little is known about how individuals with diabetes access ambulatory care. The influence of wealth on health care access and utilization of health care services, however, is an area of active research. Even within publicly funded and universally accessible systems, there is evidence that individuals from lower socio-economic groups have impaired access to care reflected in longer wait times and fewer referrals for specialist care [8][9]. This might contribute to the observation of worse health outcomes, such as the increased rate of acute diabetic complications seen in the Booth study [7], in lower income populations.

The present study is a unique examination of how SES relates to not only diabetes prevalence, but also access to diabetes care. While previous studies have documented the socio-economic gradient in diabetes prevalence and other studies have documented disparities in utilization of health care services, the present study is unique it its simultaneous examination of *both *burden of disease and utilization of health care services. We sought to determine 1) the prevalence of diabetes across income quintiles, 2) the population rates of referral to diabetes care across income quintiles, and 3) the proportion of referrals to diabetes education across income quintiles among those with diabetes. This combination of information provides unique insights into the complex interplay of burden of disease, SES and health care service use.

## Methods

### Data sources

This study used a regional Diabetes Education Centre (DEC) database that captures basic demographic information on all attendees to the regional clinic situated in Calgary, Alberta, Canada (population 928,155). The sampling frame was all active patients at the DEC from May 1, 2000 to January 9, 2002. The sample consisted of 4247 patients. All sampled patients included were from a single health region within the province of Alberta. The DEC under study is the single regional provider of diabetes education services. Access is dependent upon physician referral to the centre.

Diabetes prevalence data were obtained from the Alberta Ministry of Health and Wellness which maintains a population-based diabetes surveillance system. The Alberta surveillance system forms part of a National Diabetes Surveillance System. This system is based on administrative data and uses validated case definition algorithms to capture cases of diabetes[10,11].

Neighborhood income, education and age data were obtained from Statistics Canada Census data (2001). We defined a neighborhood as equivalent to a census dissemination area (DA). A DA is a small geographic area covered by a single census data collector and typically containing 400–700 persons. Median household incomes per DA, number of residents over the age of 65 years per DA, and number of individuals with university level education per DA were calculated. These data, along with the NDSS diabetes prevalence data were merged with the DEC database on the variable DA.

### Derivation of income quintiles

Household income quintiles were generated from DA annual income data. Each quintile contained 274 or 275 DAs. The income brackets for each quintile were as follows in Canadian dollars: quintile 1 = ≤$42781, quintile2 = $42782–$54080, quintile 3 = $54081–$64880, quintile 4 = $64881–$81017, and quintile 5 = ≥$81018.

### Study variables and analysis

Using the merged data sources, we were able to determine the following proportions: 1) The population rate of diabetes per DA (number of diabetes cases per DA/DA population); 2) the population rate of DEC referral (referral count per DA/DA population); 3) the proportion with diabetes referred to the DEC (referral count per DA/diabetes cases per DA).

Other ecologic variables were explored as possible confounders. We determined the proportion of residents with a university level education (number of respondents reporting university education per DA/DA population) as level of education is associated with income and may be inversely associated with risk for diabetes. We also determined the proportion of elderly per DA (number of respondents reporting age over 65 years/DA population) as increasing age is associated with increased risk for both low income and diabetes.

Chi square analyses were used to determine if diabetes prevalence or population rates of referral differed across income quintiles. We used Poisson regression to determine the relationship between neighborhood income, education level, and age with diabetes prevalence and referral to the DEC, controlling for education level and age. The unit of analysis in this multivariate analysis was the DA.

All statistical analyses were performed in STATA, version 8.

## Results

Population, number of diabetes cases and number of referrals by household income quintile are shown in Table 1. This table also includes the diabetes prevalence, population rate of DEC referral and the proportion with diabetes referred to the DEC per income quintile. Table 1 illustrates that diabetes cases (χ^2 ^= 743.72, *p *< 0.0005) and referrals for diabetes care (χ^2 ^= 168.435, *p *< 0.0005) were more frequent in the lower income quintiles. Referral among those with known diabetes, however, appears to be uniform across income quintiles with approximately 14% of patients with diabetes being referred to the DEC in the two year period studied. Income quintile 4 (second highest income quintile) was incidentally noted to have lower rate of referral in comparison to the other income groups (χ^2 ^= 73.9095, *p *< 0.0005).

**Figure 1:**
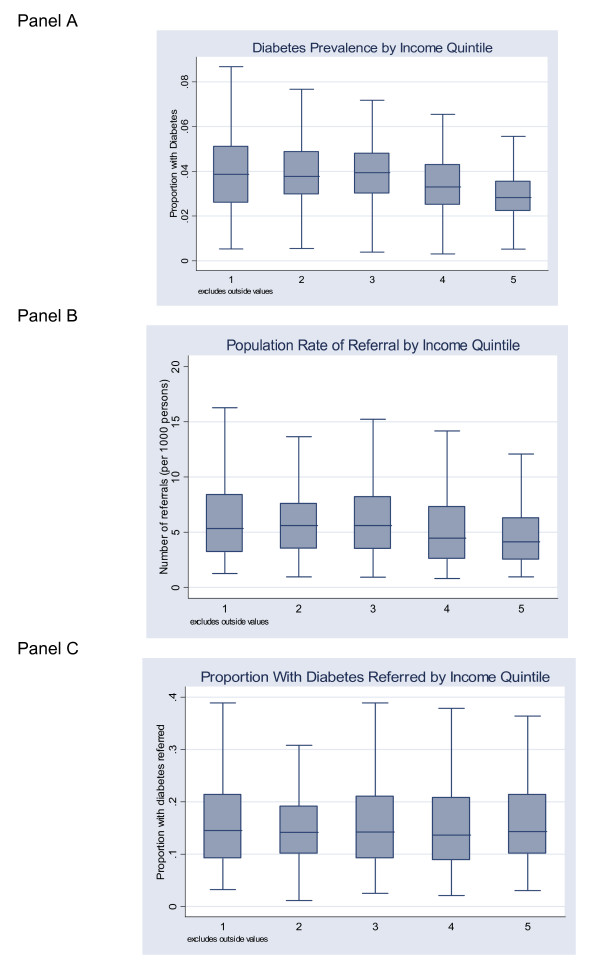
Box plots of diabetes prevalence by income quintile (panel A), population rates of DEC referral by income quintile (panel B), and proportion referred to the DEC among people with diabetes (panel C).

Figure 1 (Panel A) illustrates a box plot of diabetes prevalence across income quintiles. A socio-economic gradient is noted with the highest prevalence of diabetes in the lowest income quintiles (3.9%) and the lowest prevalence in the highest quintile (2.8%). Figure 1 (Panel B) shows the population rate of referral across income quintiles. Again, a gradient in referral is seen with the lowest quintiles having the highest rates of referral (5.3 – 5.6 per 1000 people) and the wealthy quintiles having lower rates of referral (4.1–4.4 per 1000 people). Figure 1 (Panel C) demonstrates the apparent uniformity of referral among those with known diabetes, with the proportion referred in this population remaining at approximately 14%, irrespective of income quintile.

Table 2 shows the results of the Poisson regression analysis for diabetes prevalence. This analysis reveals that the lowest income quintiles (quintiles 1, 2 and 3) had significantly higher rates of diabetes than the upper quintiles (quintiles 4 and 5). In comparison to the highest income quintile, income quintile 1 (< $42781) had a 13% higher risk of diabetes, income quintile 2 ($42782–$54080) had a 10% higher risk of diabetes and income quintile 3 ($54081–$64880) had a 15% higher risk of diabetes. Neighborhood education and age were also found to be significantly associated with diabetes risk. These latter findings indicate that if a hypothetical neighborhood had only university graduates, it would have a 78% lower rate of diabetes compared to a neighborhood with no university graduates. Similarly, in a hypothetical neighborhood with only elderly residents- the risk for diabetes would be 8 times higher than if all of a neighborhoods' residents were less than 65.

The results of the multivariate analysis examining population referral to the DEC are illustrated in table 3. Again, lower income quintiles (quintiles 1, 2 and 3) experienced a significantly higher rate of referral to the DEC compared to upper income quintiles (quintiles 4 and 5). Compared to the highest income quintile, income quintile 1 had a 33% higher rate of referral, income quintile 2 had a 28% higher rate of referral and income quintile 3 had a 31% higher rate of referral. Education was not found to be significantly associated with population rate of referral, but there was an association with age (RR = 7.62 for age > 65).

Table 4 presents the results of the multivariate analysis examining referral to the DEC among the population, controlling for prevalence of diabetes. In this model, no significant differences were found with respect to referral for diabetes care. Advanced age was predictive of a referral rate 2.4 times higher than a younger reference neighborhood. This association was present independent of income. Education, meanwhile, was not significantly associated with referral. Burden of diabetes was significantly associated with referral such that for every additional case of diabetes within a DA, there was a 1.4 % increase in the population rate of referral (RR= 1.014, 95% CI 1.013–1.015).

## Discussion

### SES & diabetes prevalence

These findings demonstrate that neighborhoods with low income have a higher prevalence of diabetes than do wealthy neighborhoods. This socio-economic gradient in diabetes prevalence has been shown previously across studies and across cultures [3,4,6]. The link between income and diabetes risk is complex. It has been speculated that the increased diabetes risk seen in low income groups is related to the increased prevalence of obesity within this group. It has already been clearly shown that low SES is associated with a much higher prevalence of obesity, especially among women [12].

Obesity remains a potent risk factor for the development of diabetes; however, low income has been shown to be an independent risk factor for the development of diabetes among women – even after controlling for body mass index and physical activity level [3]. Alternatively, low SES could be a result of diabetes in so far as disability related to diabetes complications may limit work and educational opportunities.

Neighbourhood and community level factors also contribute to the increased diabetes risk seen in low income populations. The "built" environment has been shown to be a clear barrier to physical activity in poorer neighbourhoods. Low income communities have been shown to have less biomass and park-space compared to wealthier communities [13]. There may also be a perception that it is less safe to walk in a poorer neighbourhood – this not only deters physical activity but erodes the sense of community among residents [3,13,14]. This sense of community, along with established social networks, has been shown to be protective against certain negative health outcomes [3].

### SES and diabetes care utilization

Previous studies examining the association of income on access and/or utilization of health care services have suggested that even within single payer systems such as Canada's, access may not be universal. Dunlop and colleagues showed that poor individuals are more likely to visit their family physician, but that the wealthy are nearly twice as likely to be referred on to specialty care [8]. The wealthy are also likely to have a shorter wait time for procedures such as coronary angioplasty [9].

Consistently reaching therapeutic targets in diabetes usually requires the support of a multidisciplinary team (diabetes educators, registered dieticians, and social worker and diabetes medical specialists) and the use of several medications [15-17]. Diabetes education centres allow patients to access the relevant health care professionals and education services within a single centre [15]. Previously, little was known about how individuals access such diabetes care services in this centralized model of care, particularly in relation to that individual's socio-economic standing.

The present study shows that people in the lowest income strata were more likely to be referred for structured diabetes education and care. Our study shows that low income patients are approximately 30% more likely to be referred to this DEC and that this seems to appropriately reflect burden of disease.

### SES and utilization of diabetes care controlling for prevalence of diabetes

Our unique ability to study DEC access while also knowing prevalence of diabetes indicates that referral of patients with diabetes is quite consistent across income quintiles. Therefore, the utilization gradient seen truly reflects disease burden and implies that there is no access bias based on income. This is a positive finding but somewhat surprising in light of a history of studies suggesting that less affluent individuals have impaired access to care. We speculate that increasing patient awareness of the "diabetes epidemic" may be leading to more patients requesting referral. It is also possible that the DEC may be viewed as an extension of primary care. Low income populations have a higher burden of health problems in general, and the primary care physicians who serve these communities may view the DEC as the most efficient way to provide complex patients (ie those with diabetes) with the care they require. Given the finding of Dunlop and colleagues [8] of good access to primary care for lower income individuals, it is perhaps not that surprising that less affluent patients who are visiting their family physician frequently are also accessing the DEC.

Primary care physicians' threshold for referral warrants further examination. It was notable that among those with diabetes, the level of general education was significantly associated with referral. It is possible that better educated patients are better advocates for their health and as a result perhaps more likely to be referred earlier in the natural history of their condition. While we did not see a socioeconomic gradient in overall access to the DEC, we cannot exclude the possibility that wealthy individuals may have been referred earlier and with less co-morbid disease.

This study is limited by its cross-sectional nature. We also examined the association of SES and referral in the context of a centralized model of diabetes care. Our findings, while applicable to the health region and period under study, may not be indicative of how services are utilized elsewhere. It should be noted that the city under study is relatively wealthy. Only 6% of this study population lived at or below the national poverty line. However, post-hoc analyses demonstrated that while there was a higher prevalence of diabetes among those who live in poverty, access to diabetes care was not significantly different to those with higher income. This study also examined data aggregated to the level of dissemination area, and therefore was ecologic in nature. We must always be cautious when making inferences about individuals when a study examines a grouping of individuals (e.g., individuals living in a geographic area) rather than the individuals themselves (i.e., the ecologic fallacy). While finding that low income was associated with diabetes and referral to diabetes care, we cannot say that it was indeed the lowest income individuals in these neighborhoods that were the most likely to have diabetes or to be referred. It is plausible that within low income groups, it was those with relatively higher earnings that accessed care. Finally, the validity of using neighborhood income as a surrogate for individual level income has been called into question by previous studies [18-20]. There is emerging evidence, however, that neighborhood-level income is in and of itself an independent SES construct that is a valid predictor of health outcomes, over and above any effect relating to individual income [21].

In spite of these limitations, the present study provides encouraging data that diabetes care services are being accessed and utilized by those who require it. This study involved a unique combination of several data sources, and with this richness of data was able to show that while diabetes may be more prevalent in lower income regions, individuals living in such regions appear to be as successful at accessing diabetes care services as their wealthier counterparts.

## Competing interests

The author(s) declare that they have no competing interests.

## Authors' contributions

All of the listed authors contributed to the presented study and meet the criterion for authorship. DMR and WAG conceived and designed the study. WAG, ALE, PN, PMS, ETL developed the regional database used in the presented study. LWS contributed data from the National Diabetes Surveillance System. DMR performed the statistical analyses and DAS assisted with the analysis and provided statistical expertise. DMR wrote the manuscript but all of the listed authors were actively involved in the editorial process and provided approval of the final, submitted manuscript.

## Pre-publication history

The pre-publication history for this paper can be accessed here:



## Figures and Tables

**Table 1:** Characteristics of Income Quintiles

Income Quintile	Population	DA Count	DM Cases	Number of referrals	Diabetes Prevalence* (percent)	Population rate of referral** (per 1000)	Proportion with diabetes referred^§ ^(percent)
1 (lowest)	157 020	274	6521	926	3.9	5.3	14.5
2	163 485	275	6501	859	3.7	5.6	14.2
3	174 010	274	7049	907	3.9	5.6	14.2
4	214 630	274	6956	805	3.3	4.4	13.6
5 (highest)	219 010	274	6243	788	2.8	4.1	14.3

* Median diabetes prevalence per DA

**Median population rate of referral per DA

^§ ^Median proportion with diabetes referred per DA

**Table 2:** Poisson Model for Diabetes Prevalence

	Incidence Rate Ratio	95% Confidence Interval	*p*-Value
Income Quintile			
1 (lowest)	1.13	1.09–1.17	< 0.0005
2	1.1	1.06–1.14	< 0.0005
3	1.15	1.11–1.19	< 0.0005
4	1.01	0.97–1.04	0.722
5 (highest)	1.0 (reference)		
University Education	0.22	0.20–0.25	< 0.0005
Age > 65	8.23	7.32–9.26	< 0.0005

**Table 3:** Poisson Model for Referral to the Diabetes Education Centre among the General Population

	Rate Ratio	95% Confidence Interval	*p*-Value
Income Quintile			
1 (lowest)	1.33	1.2–1.48	< 0.0005
2	1.28	1.15–1.42	< 0.0005
3	1.31	1.18–1.45	< 0.0005
4	1.06	0.96–1.18	0.228
5 (highest)	1.0 (reference)		
University Education	0.86	0.63–1.17	0.332
Age > 65	7.62	5.52–10.52	< 0.0005

**Table 4:** Poisson Model for Referral to the Diabetes Education Centre, Controlling for Diabetes Prevalence

	Rate Ratio	95% Confidence Interval	*p*-Value
Income Quintile			
1 (lowest)	1.07	0.96–1.18	0.21
2	1.05	0.94–1.17	0.372
3	1.05	0.95–1.17	0.319
4	1.05	0.95–1.16	0.345
5 (highest)	1.0 (reference)		
University Education	1.19	0.87–1.61	0.276
Age > 65	2.39	1.70–3.37	< 0.0005
Diabetes Cases	1.014	1.013–1.015	< 0.0005
